# Implications of Nubian-Like Core Reduction Systems in Southern Africa for the Identification of Early Modern Human Dispersals

**DOI:** 10.1371/journal.pone.0131824

**Published:** 2015-06-30

**Authors:** Manuel Will, Alex Mackay, Natasha Phillips

**Affiliations:** 1 Department of Early Prehistory and Quaternary Ecology, University of Tubingen, Tübingen, Germany; 2 Centre for Archaeological Science, School of Earth and Environmental Sciences, University of Wollongong, Wollongong, Australia; University of Oxford, UNITED KINGDOM

## Abstract

Lithic technologies have been used to trace dispersals of early human populations within and beyond Africa. Convergence in lithic systems has the potential to confound such interpretations, implying connections between unrelated groups. Due to their reductive nature, stone artefacts are unusually prone to this chance appearance of similar forms in unrelated populations. Here we present data from the South African Middle Stone Age sites Uitpanskraal 7 and Mertenhof suggesting that Nubian core reduction systems associated with Late Pleistocene populations in North Africa and potentially with early human migrations out of Africa in MIS 5 also occur in southern Africa during early MIS 3 and with no clear connection to the North African occurrence. The timing and spatial distribution of their appearance in southern and northern Africa implies technological convergence, rather than diffusion or dispersal. While lithic technologies can be a critical guide to human population flux, their utility in tracing early human dispersals at large spatial and temporal scales with stone artefact types remains questionable.

## Introduction

Similarities in material culture among populations can arise by three pathways: convergence (independent innovation), dispersal (movement of people) or diffusion (movement of ideas/objects or cultural exchange). Historically, most similarities in material culture between two or more samples have been explained through the latter two mechanisms [1–10], though the problem of convergence has long been appreciated [11–13]. Dispersal denotes the physical movement, migration or relocation of a group of people from one area to another together carrying with them (parts of) their material culture, while cultural diffusion involves the movement of ideas or objects from their place of origin to another population in a different area via information transmission (e.g. cultural exchange, stimulus diffusion, trade etc.) without associated movement of people [3,6,14–17].

In contrast to diffusion and dispersal, convergence describes similar ideas or objects arising from independent innovation, analogous to homoplasy of character states in evolutionary biology [18–25]. Such ideas and objects cannot be used to resolve historical relationships between populations and cultures, just as homoplasies do not provide phylogenetic information on organisms [18–25]. The problem of convergence in material culture is particularly acute in lithic technologies, an essentially reductive method of tool manufacture bound by functional requirements of edge production, and by the physics of fracture mechanics [26–28]. These parameters constrain the potential effective morphological space and increase the likelihood of reaching the same form independently (cf. [24,25] for an analogous argument regarding homoplasies in biological systems). From an empirical point of view, convergence can be demonstrated by the fact that similar kinds of lithic artefacts, such as Levallois cores, bifacial lanceloate points, tanged tools or backed microliths, were independently developed by populations widely spread in space and time, precluding *a priori* assumptions of shared information systems [29–32]. Nevertheless, the unique durability of lithic artefacts and their tendency to pattern in space and time means that they remain the basis for most assessments of population flux in the Palaeolithic. This is particularly the case where ancient human DNA is as yet unavailable and given that modern DNA alone can more easily isolate population changes in time than in space [33,34]. More specifically, the identification of past populations with certain lithic assemblages has been consistently employed in the interpretation of human dispersals within, out of, and beyond Africa (e.g., [8,10,35,36–43]).

Dispersal, diffusion and convergence are likely to produce different signals in the archaeological record. Because they involve the retention and movement of information, dispersal and diffusion are consistent with an archaeological pattern of continuity in the occurrence of technological elements through contiguous ranges of space and time. Dispersals should leave an additional genetic signature in the populations involved (cf. models of the spread of farming to Europe [6,14,15,44–46]). The technological element under study itself offers another dimension to distinguish between diffusion and dispersal: cultural diffusion likely operates through product copying and thus the lower-fidelity transmission of more simple assemblage elements, while dispersal would involve process copying and the relatively high-fidelity transmission of more derived systems [41,47–51].

Technological convergence produces different archaeological expectations to diffusion and dispersal. Independent innovation in two unrelated populations is implied by a) a large gap in the spatio-temporal distribution of the technological elements under consideration, and b) replication of only a limited subset of the technological repertoire in the two separated assemblages. Furthermore, as the range of space and time considered increases, so does the probability of convergence, particularly where the technologies are nested in a shared ancestral system [30]. Convergence is a parsimonious explanation where a single assemblage element replicates between spatio-temporally separated samples across a large spatial range—a problem that in particular has plagued the search for inter-continental transmission [40].

In order to limit the confounding potential of convergence, research in the Palaeolithic has usually focused on the most derived components of lithic assemblages, where elaborate, multi-step flaking systems reduce the probability of chance morphological similarities. In more basic elements of lithic technology, such as retouched flakes, equifinality of form is almost inevitable [32,52]. In this respect, systems of core reduction, which include long sequences of interdependent actions, have been a particular focus (e.g. [36,41,53,54]).

## The Nubian Techno-Complex and Early Modern Human Dispersals

The Nubian techno-complex provides a significant recent example of attempts to trace broad-scale movements of populations and ideas through a specific core reduction system and its associated products. The Nubian system is a tightly-defined subset of the preferential Levallois core reduction method, in which flakes and points of predetermined size and form are manufactured [55]. Due to its elaborate and specific method of core preparation, the Nubian techno-complex has been equated both with an information sharing network [56] and a group of people [37,57,58] (though note [59]).

Other than the presence of Nubian core technology and the concomitant production of points, proponents have not reached consensus on what additional technological and typological criteria define this cultural unit or how frequent Nubian cores need to be for an assemblage to be characterised as Nubian [56–58,60–64]. This disagreement foregrounds the presence of cores as the principal binding element of Nubian assemblages, but inhibits more complex comparisons between regions [59,65–71].

Based primarily on the presence of Nubian cores, the spatial distribution of the Nubian techno-complex was initially limited to north-east Africa, and age-constrained to MIS 5 (130–74 ka). While proportionally few Nubian sites have been well-dated (cf. [63,66,71]), researchers have distinguished an ‘early Nubian techno-complex’ dating to early MIS 5 (i.e. MIS 5e, ~120 ka), characterized by an emphasis on bilaterally prepared Nubian type 2 cores and a bifacial façonnage component (see further discussion below). In contrast, a ‘late Nubian techno-complex’ is said to feature a higher a proportion of Nubian Type 1 cores with distal divergent preparation but without bifacial elements, dating to the second half of MIS 5 [37,39,57,58,64,72,73]. This ‘late Nubian techno-complex’–or N-group [56,61]–has sometimes been seen as the Nubian techno-complex *sensu stricto*, belonging mainly to MIS 5a [39,64,73].

Various usage sees the distribution of the Nubian techno-complex range from specific parts of Northeast Africa, to the combined regions of northern Sudan, the middle and lower Nile Valley, the Eastern Sahara and the Red Sea Mountains and through to “Northeast Africa” more generally [37,39,56,64,73]. Occasional cores with Nubian characteristics have also been reported from Ethiopia [74–77], Kenya [78], Somalia [75], Libya [69], Algeria [70] and as far west as Mauritania [79]. While the majority of reports place the Nubian techno-complex into MIS 5 and thus older than 74 ka [37], the chronological range of Nubian cores extends into MIS 4 and potentially early MIS 3 at the site of Taramsa 1 [73]. That being said, this single MIS 3 occurrence has been referred to the Taramsan rather than the Nubian techno-complex [64,72,73], with volumetric blade debitage replacing Nubian point core reduction.

Recently, documentation of Nubian cores in southern and central Arabia has been used to infer ‘demographic exchange across the Red Sea’, and the concerted presence of anatomically modern humans in the region before 100 ka [37,57,58]. This would represent one of the earliest identified populations of African-derived modern humans outside of Africa, and has been used to support arguments for the southern migration route of modern humans into Asia and Oceania [57]. This hypothesis maintains that the “Afro-Arabian Nubian techno-complex” in both North-East Africa and the Arabian Peninsula, defined on the presence of Nubian cores, reflects the same group of people using this specific reduction technology [37,57,58]. While the chronological control over many sites is weak—particularly on the Arabian Peninsula where they occur almost exclusively as surface assemblages [66]–the slightly later presence of Nubian cores in Arabia is interpreted as evidence for modern human dispersals from their source area in northeast Africa. Two cores with Nubian characteristics have also been reported from the Thar Desert in India and are tentatively associated with early modern human dispersals [80].

Our interest in this paper is whether current definitions preclude the classification of cores from distant parts of Africa as Nubian, and whether any identified similarities more likely arise from dispersal, diffusion or convergence. This has a bearing on the certainty with which the Afro-Arabian Nubian techno-complex can be used to support arguments for anatomically modern humans outside of Africa by 100 ka, for the support that the Afro-Arabian Nubian techno-complex offers the identification of dispersal routes, and also for a number of other arguments concerning early dispersals of modern humans within and beyond Africa [8,36,40,41,81].

### Defining the Nubian core reduction system

**Fig 1:**
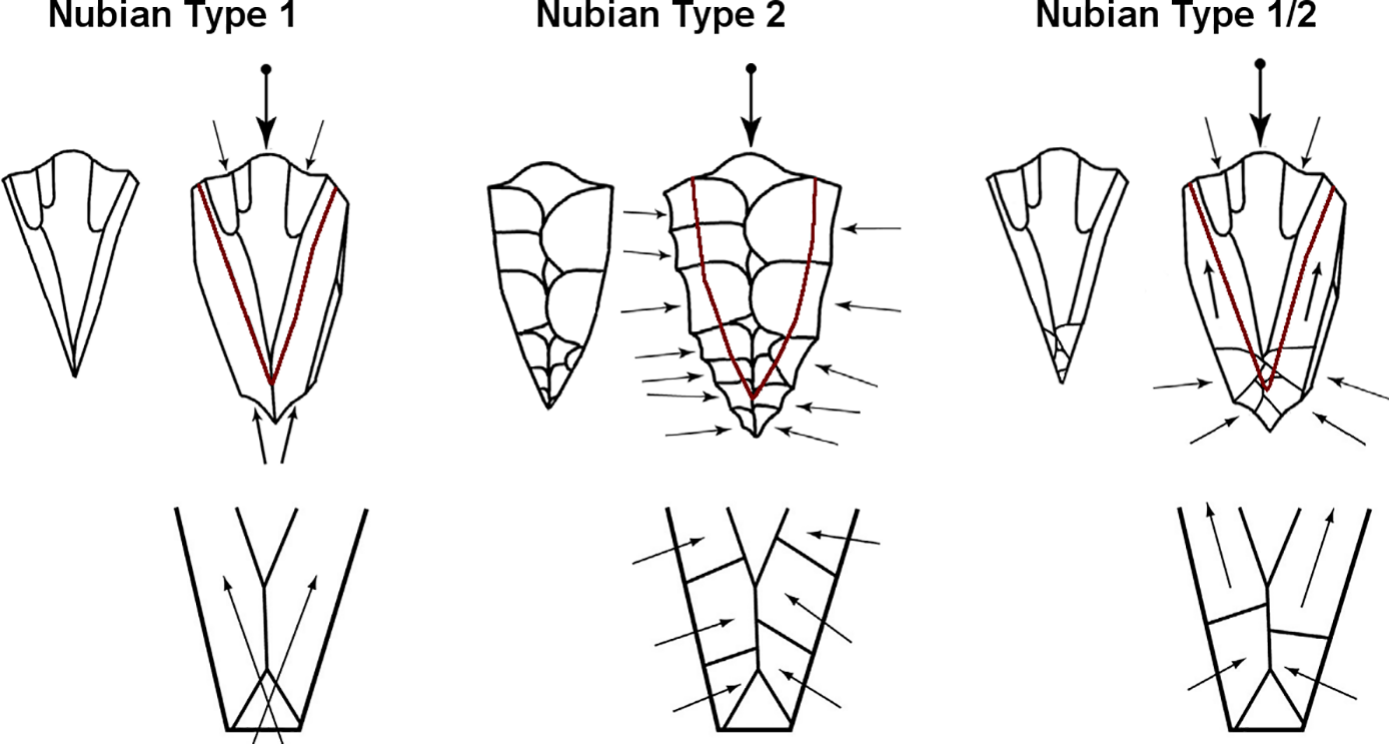
Schematic depiction of the three Nubian core types following [58].

Formal definitions of the Nubian core reduction system are provided by [55,57,58,60]. Initial definitions delimited two distinct Nubian methods [55,60], though the discreteness of those classes is disputed [82]. Recent definitions [57,58] recognise three Nubian core types (Fig 1), with the potential for transformation between them during the reduction of a single core [82]. Nubian type 1 cores involve the production of a distal ridge by two divergent debordant removals from the distal platform. Type 2 cores involve production of a distal ridge by a series of centripetal removals from the lateral margins of the core. Type 1/2 cores involve a combination of distal and lateral removals to establish the distal ridge that controls final flake form. For all of these core forms, convergent flakes (or points) constitute the main end products.

Usik et al. [58] present four necessary technological attributes for a core to be classified as Nubian: a steeply angled median distal ridge <120° and generally >60°; an opposed striking platform with angle of intersection to the exploitation surface varying from 50–90°; a triangular core shape (including triangular, cordiform, and pitched); and a prepared main striking platform. They state that “such a rigid definition is necessary to prevent any unwarranted broadening of this particular reduction strategy” (p. 249). As a further criterion, one can add the existence of one or more main convergent removals on the core´s primary working surface indicating the exploitation of Nubian cores for desired end products. While size does not form part of any definitions of the Nubian system, Usik et al. [58] distinguish cores less than 80 mm in length as ‘micro-Nubian’. Their data suggest a continuous, if not evenly distributed, set of cores lengths from 40 mm to 180 mm.

## Material and Methods

### Uitspankraal 7

**Fig 2:**
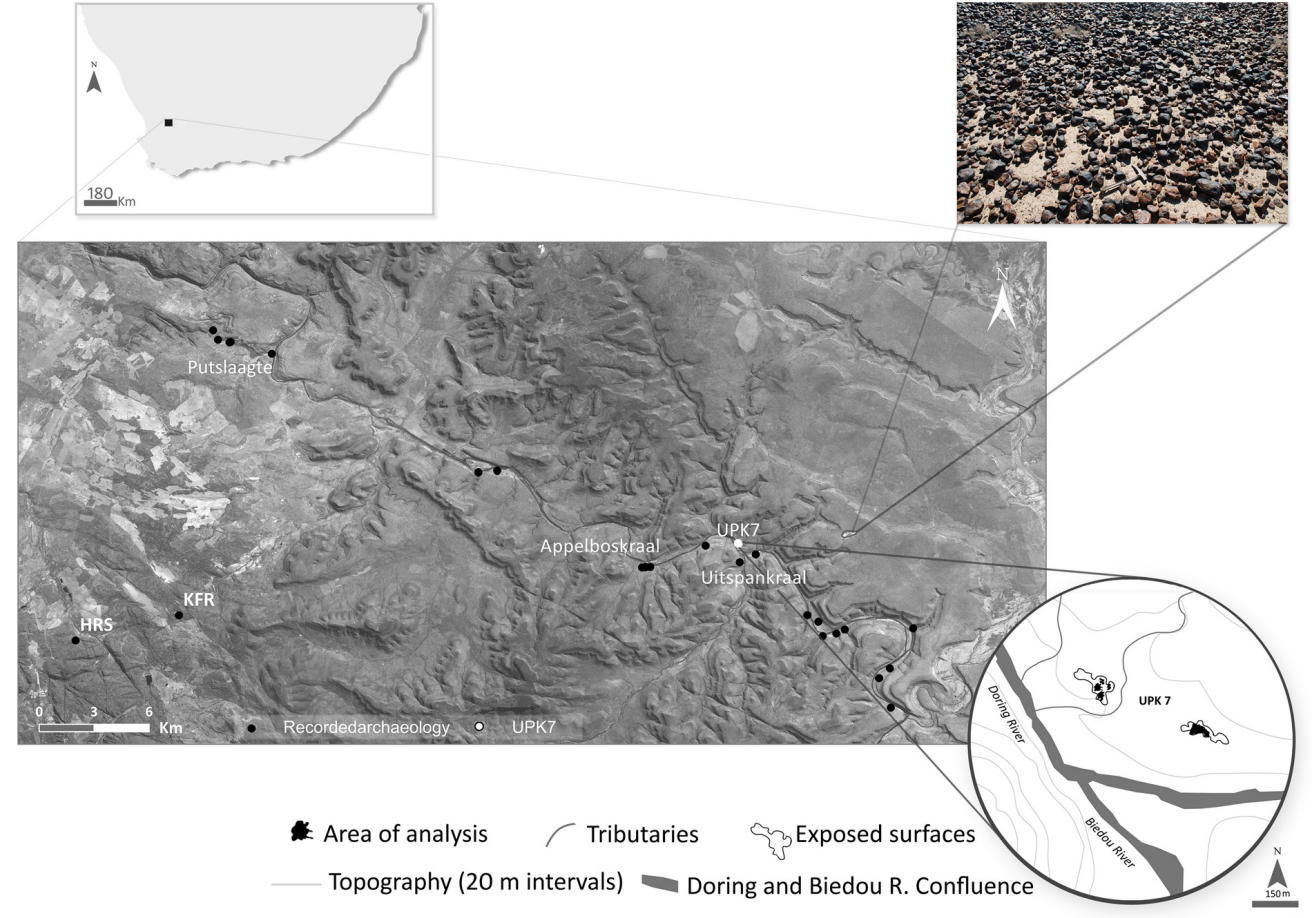
Location of UPK7 and nearby sites in southern Africa, including Mertenhof (MRS), Hollow Rock Shelter (HRS) and Klipfonteinrand (KFR). Inset (top right) are silcrete cobbles from Swartvlei. Landsat panchromatic EDM+ scene downloaded from Global Land Cover Facility (University of Maryland) at http://glcf.umiacs.umd.edu/index.shtml. Original scene sourced and modified from [125].

**Fig 3:**
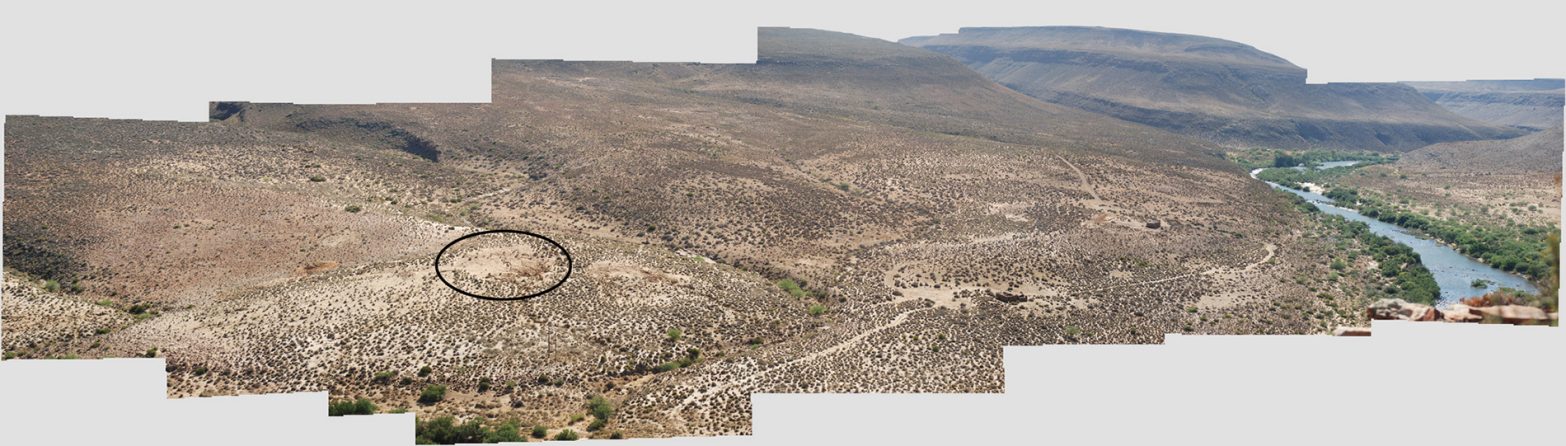
Panoramic view of UPK7 (black circle) adjacent to the Biedouw River.

Our principal data for this paper derive from the open air site Uitspankraal 7 (UPK7), situated at the confluence of the Doring and Biedouw rivers in south-western South Africa (Figs 2 and 3). Unlike many parts of Africa, the regional sequence of lithic technological changes in southern Africa is reasonably well-documented, and generally well-dated [41,83,84]. We concentrate here on the Middle Stone Age (MSA) parts of that sequence (Table 1), as it is during the MSA that the Afro-Arabian Nubian techno-complex occurs. Though the MSA of southern Africa shares characteristics with that of north-eastern Africa, including the use of Levallois and discoidal modalities, no Nubian Levallois has previously been identified (though note [85] figs 29 & 30), consistent with the spatially circumscribed notion of the Nubian techno-complex.

**Table 1:** Characteristics of major Middle Stone Age industries in southern Africa, following [41, 83, 84, 92–94].

Industry	Age (ka) (approx.)	Major implements	Blank types	Raw material selection
Late MSA	30–50	None known	Points, flakes	Local
Post-Howiesons Poort	50–60	Unifacial points, scrapers	Levallois points, blades	Some preferential selection for silcrete
Howiesons Poort	60–65 (60–110)	Backed artefacts, notched blades	Blades	Heavy preferential selection for silcrete
Still Bay	70–80 (70–110)	Bifacial points	Bifacial thinning flakes	Some preferential selection for silcrete
Early MSA	>80	Denticulates	Levallois points, long blades	Local

We restrict the industries described to those occurring in the modern winter and year-round rainfall zones, given potential issues of comparability with industries from further away [41]. Ages in brackets are based on the sequence of Diepkloof Rockshelter [95], which has so-far yielded a uniquely older signal (but see [92, 96]).

**Fig 4:**
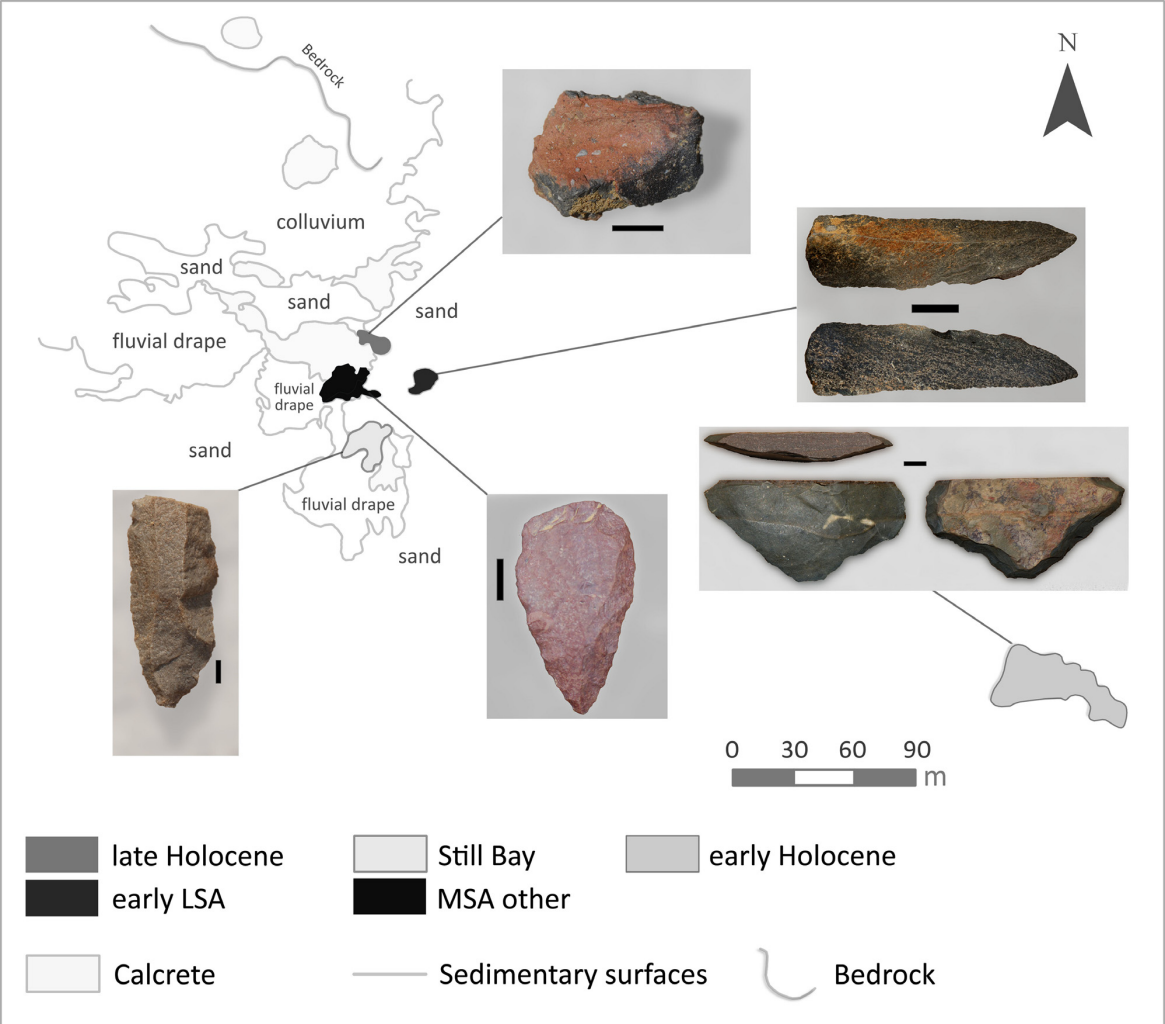
Overview of the UPK7 surface scatter indicating the distinct spatial patterning of time-sensitive cultural elements.

UPK7 is a dense scatter of flaked, ground and battered stone artefacts situated ~7 m above the Doring River (Fig 3). The site is actively eroding, with artefacts from multiple palaeosols deflated onto and migrating across the present surface. In spite of this, the site retains spatial structure in many of its time-sensitive elements, with distinct clustering of late Holocene (pottery), early Holocene (naturally backed knives), early Later Stone Age (small blades and platform cores), Still Bay (bifacial points), other MSA (discoidal and Levallois cores; denticulates) and Acheulean (handaxes) markers (Fig 4).

In total we analysed and mapped 9350 artefacts from UPK7 during the field season conducted in 2014. All artefacts were assigned unique identification numbers, analysed in situ and plotted individually in the WGS 84 coordinate system with a total station, using local control points established with a Trimble RTK base and rover DGPS. As we performed in-field analyses on the UPK7 materials only, without collecting or displacing any artefacts, no permits were required. Heritage Western Cape (HWC) requires permits only for the collection and destruction of archaeological material. For the present study, all artefacts had their spatial location recorded to within 1 mm, were analysed in-field by non-destructive methods, and were then returned to their original location. Permission to access the farm Uitspankraal was obtained through Manus and Lillie Hough, owners of Uitspankraal farm.

The sampling included analysis of time-sensitive artefacts identified across the site during repeated non-systematic sampling over one month, and a complete sample of artefacts >20 mm in two areas comprising 299 m^2^, or 3.9% of the total site area. One of these areas comprising 21 m^2^ appeared to consist principally of an early Later Stone Age (LSA) variant [86], the other contained of a mix of MSA components including an accumulation of what we believed to be post-Howiesons Poort artefacts (278 m^2^). This area and its immediate buffer were the main focus of our seasons’ work.

### Main area methods and sample

The following methods of lithic analysis were employed in the systematic sampling of the main area of UPK7. Key data captured for all artefacts >20 mm were: raw material, artefact class (flake, retouched flake, core, flaked piece), completeness, artefact type (including tool type or core type), reduction system, weight, maximum dimension, cortex %, cortex type, weathering and reworking. We collected metric data of lithic artefacts using analogue callipers accurate to 1 mm and electronic scales with minimum 1 g precision. All data were entered into Lenovo tablets by a recorder working with an analyst.

Additional attributes were recorded on all Levallois cores with Nubian-like characteristics following the definitions and recommendations of [58]. All attributes and measurements were taken by AM and MW and cross-checked to ensure replicability. We measured angles with a goniometer to the nearest degree (°) and dimensions with analogue callipers to the nearest millimetre (mm). In addition to the attribute analysis, each core was photographed and schematically sketched on a spread sheet in the field to record the configuration and sequence of removals on the main working surface (S1 Text).

While we made no systematic attempt at refits during our analysis, we nevertheless identified three refit sets variously comprising three complete flakes, two complete flakes, and one flake and core set. We also conjoined broken artefacts in a further four locations. These observations reaffirm the spatial integrity of the site suggested by the distinct clustering of time-sensitive cultural elements.

### Non-systematic samples

Over the course of October 2014 we repeatedly walked the erosional area surrounding the main analytic area at UPK7. During this time we flagged any observed artefacts with potential time-sensitive characteristics. These included backed microliths (n = 2), bifacial points (n = 9), unifacial points (n = 3), denticulates (n = 7) and pieces of pottery (n = 15), as well as Levallois cores with Nubian-like characteristics. These artefacts were all mapped and analysed at the end of the season. Necessarily these results are not exhaustive but serve to contextualise and in some cases supplement the main analytic sample.

### Mertenhof

**Fig 5:**
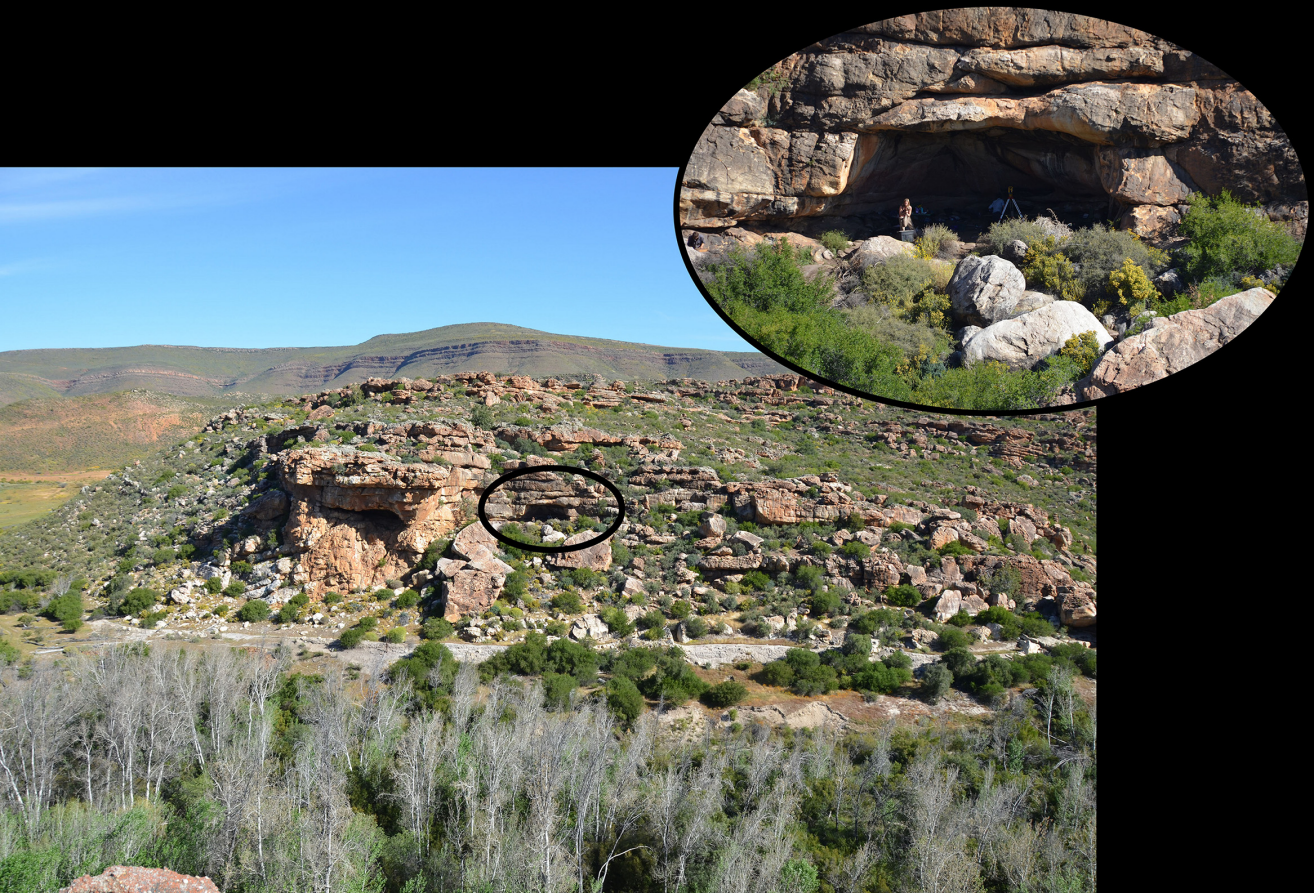
View of the Mertenhof archaeological site with focus on the rock shelter entrance.

In addition to UPK7, we present data from ~11800 piece plotted artefacts from the site of Mertenhof (MRS), a rock shelter located 25 km away on the Biedouw River. Three seasons of excavation have been undertaken at MRS so far under the direction of AM and Aara Welz (Fig 5; see S2 Text). The research permit to conduct archaeological excavations at Mertenhof is issued under the National Heritage Resources Act (Act 25 of 1999) and the Western Cape Provincial Gazette 6061, Notice 298 of 2003 and valid from April 2013–2016. AM is the permit holder (permit number: 130306TS13).

All recovered lithic artefacts are temporarily housed in the Department of Archaeology at the University of Cape Town pending accessioning at Iziko South Africa Museum, 25 Queen Victoria Street, Cape Town, 8001, South Africa, where they will be available for further analysis. Mertenhof specimen numbers range from 1–12923 (season 1), 20000–29429 (season 2) and 30000–42600 (season 3).

Mertenhof is one of seven MSA sites excavated within 100 km of UPK7: Elands Bay Cave, Diepkloof, Varsche Rivier 3, Klein Kliphuis, Putslaagte 1, Putslaagte 8 and Klipfonteinrand being the others [86–91]. These assemblages allow the UPK7 finds to be situated in the regional technological and chronological sequence.

## Results

**Fig 6:**
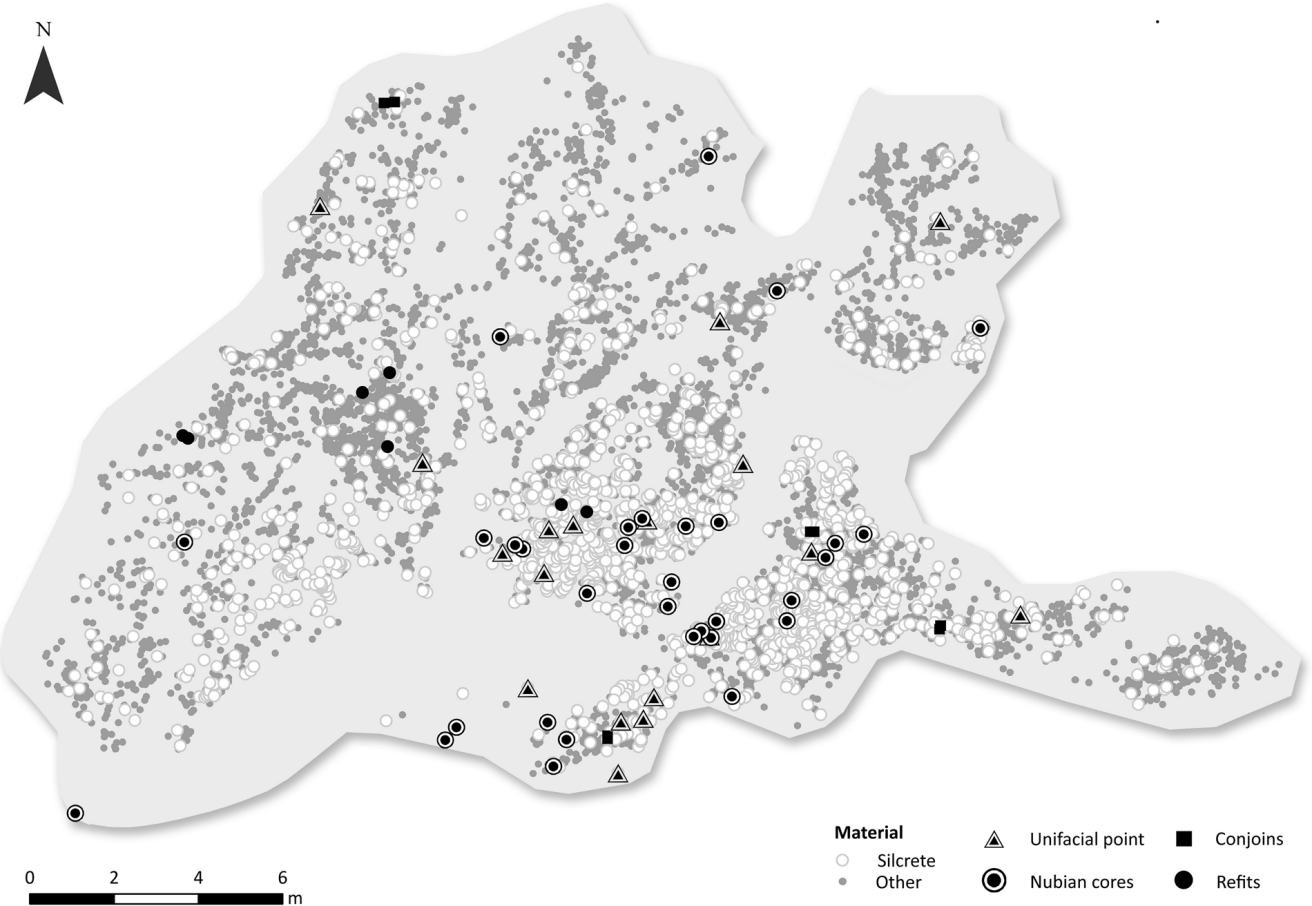
Distribution of artefacts in the main area at UPK7, with a focus on silcrete (white circles), unifacial points (nested triangles), and cores with Nubian characteristics (nested circles).

**Fig 7:**
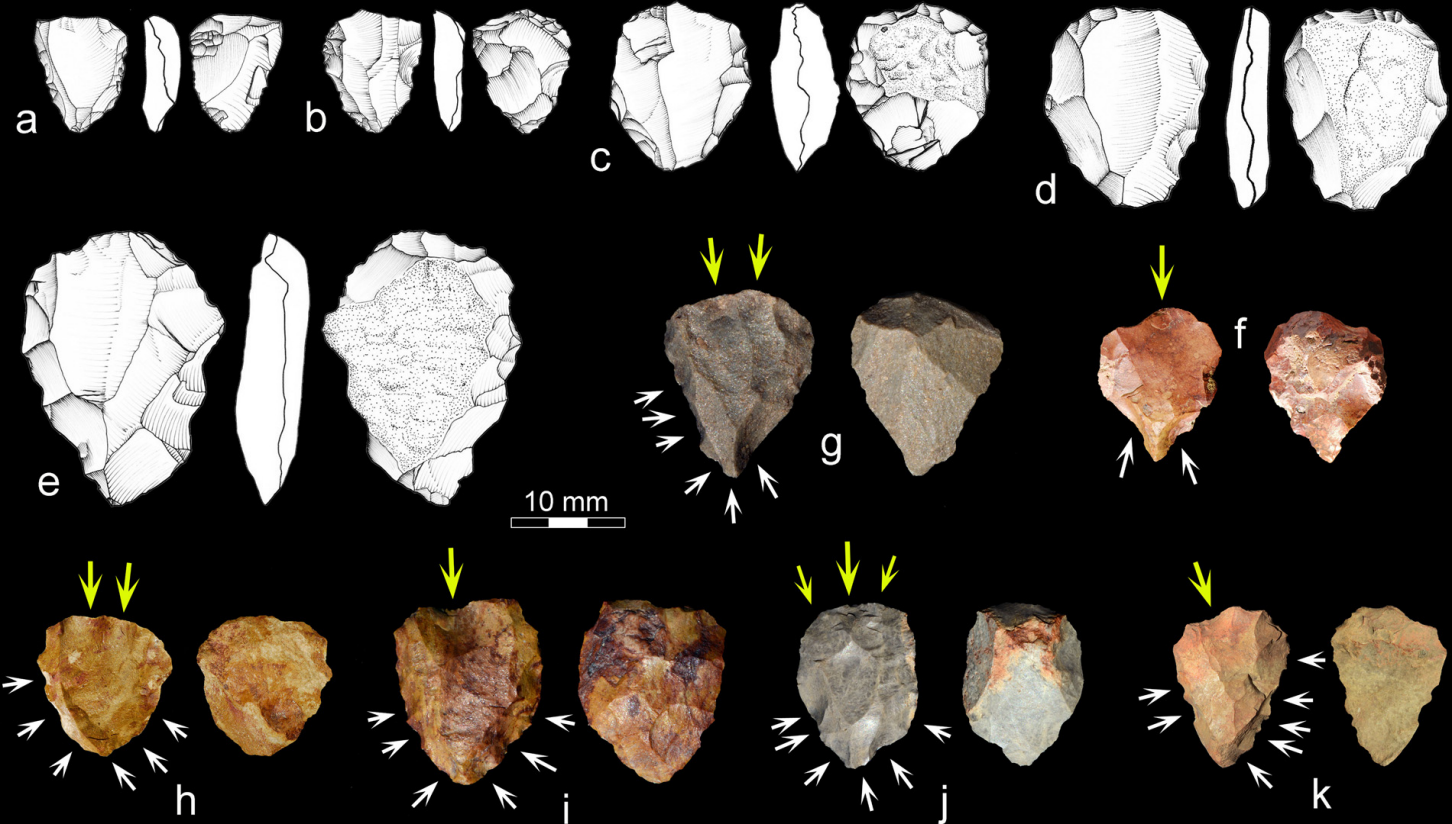
Cores with Nubian characteristics from UPK7 and Mertenhof. UPK7: a, c, h, f & i, silcrete; b & j, chert; d, hornfels; e & g, quartzite. Mertenhof: k, silcrete. White arrows on photos show direction of lateral and distal preparation; yellow arrows show direction(s) of preferential blows.

The distribution of artefacts at UPK7 reveals distinct high density clustering in the south east quadrant of the analysed area (Fig 6). This high density area is notably silcrete-rich. Fourteen unifacial points were recorded in this area, while a further four were identified in the lower density fringes of the main concentration. Here and below, we refer to all convergent flakes (or point blanks) with dorsal-only retouch as unifacial points, as is common in southern African nomenclature. We mapped and analysed 31 preferential Levallois cores with possible Nubian characteristics in this main sample area (Figs 6 and 7), with a further five such cores being identified in surrounding parts of the site.

We analyzed the 36 potential Nubian cores in terms of the stringent technological definition provided above by [58] to preclude misidentifications. These cores conform either to type 1/2 (58%) or type 2 (36%) variants of the Nubian system (Fig 7), with only one specimen exhibiting type 1 preparation. Knappers manufactured these cores on all principal raw materials at UPK7, including silcrete, quartzite, hornfels and chert. Most of the cores are on silcrete (56%), consistent with their presence in the silcrete-rich area of the site.

The characteristics of the Nubian-like cores at UPK7 conform to the strict technological taxonomic classification by [58] (Table 2). More than 97% of cores (35 of 36) show a steeply angled median distal ridge that serves to control the distal lateral convexity of the core’s primary working surface (mean = 87.2°; range = 59°-135°). Most cores (61%) fall into the “semi-steep” category of [58]. In all cases, knappers set up an opposed secondary striking platform for the preparation of the distal ridge, with a narrow distribution of distal platform angles (mean = 68.7°, range = 53–82°; 82% “semi-acute”). The main and distal striking platforms were treated differently and independently. Knappers always prepared the lateral core margins before installing the distal striking platform.

**Table 2:** Summary statistics of metrics taken on the Nubian cores from UPK7.

	Median distal ridge angle (°)	Distal platform angle (°)	Weight (g)	Maximum dimension (mm)	Length (mm)	Width (mm)	Thickness (mm)	Last removal length (mm)
Mean	87.2	68.7	31.2	45.8	44.4	36.7	16.5	35.4
Min.-Max.	59–135	53–82	8.3–123.1	35–88	33–86	27–66	8–32	10–69
SD	15.0	7.9	28.3	11.6	11.6	9.0	5.7	12.1
n	32	33	34	34	33	33	33	31

The Nubian-like cores at UPK7 correspond in 91% to a triangular core morphology, which is most often cordiform (48%). The main striking platform is always prepared, with a dominance of facetted (60%) over dihedral (40%) butts. In terms of end products, most of the cores in our sample exhibit one or more convergent removals on the core´s primary work surface (91%), with flake and blade negatives being rare.

**Fig 8:**
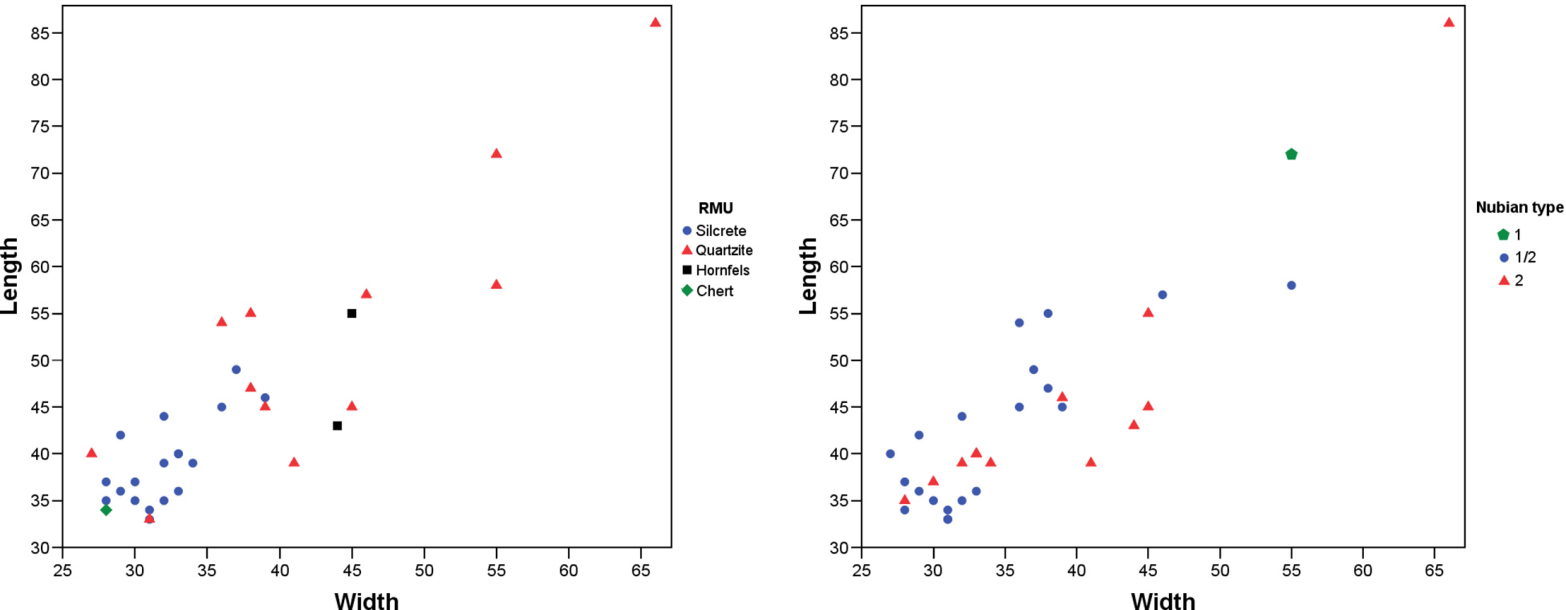
Scatter plots showing length by width (in mm) for Nubian-like cores from UPK7 with indication of raw material (left) and Nubian core type (right).

The Nubian-like cores at UPK7 are small relative to those from north-eastern African and Arabia (mean length = 44.4 mm, range: 33–86 mm; Fig 8; Table 2), but generally fall in the lower end of the size spectrum of Nubian cores in [58]. The difference in size is likely driven by available raw materials. The silcrete in the sample probably derives from Swartvlei, 5 km SE of the site, where the rock occurs in the form of small nodules <100 mm (Fig 2).

**Fig 9:**
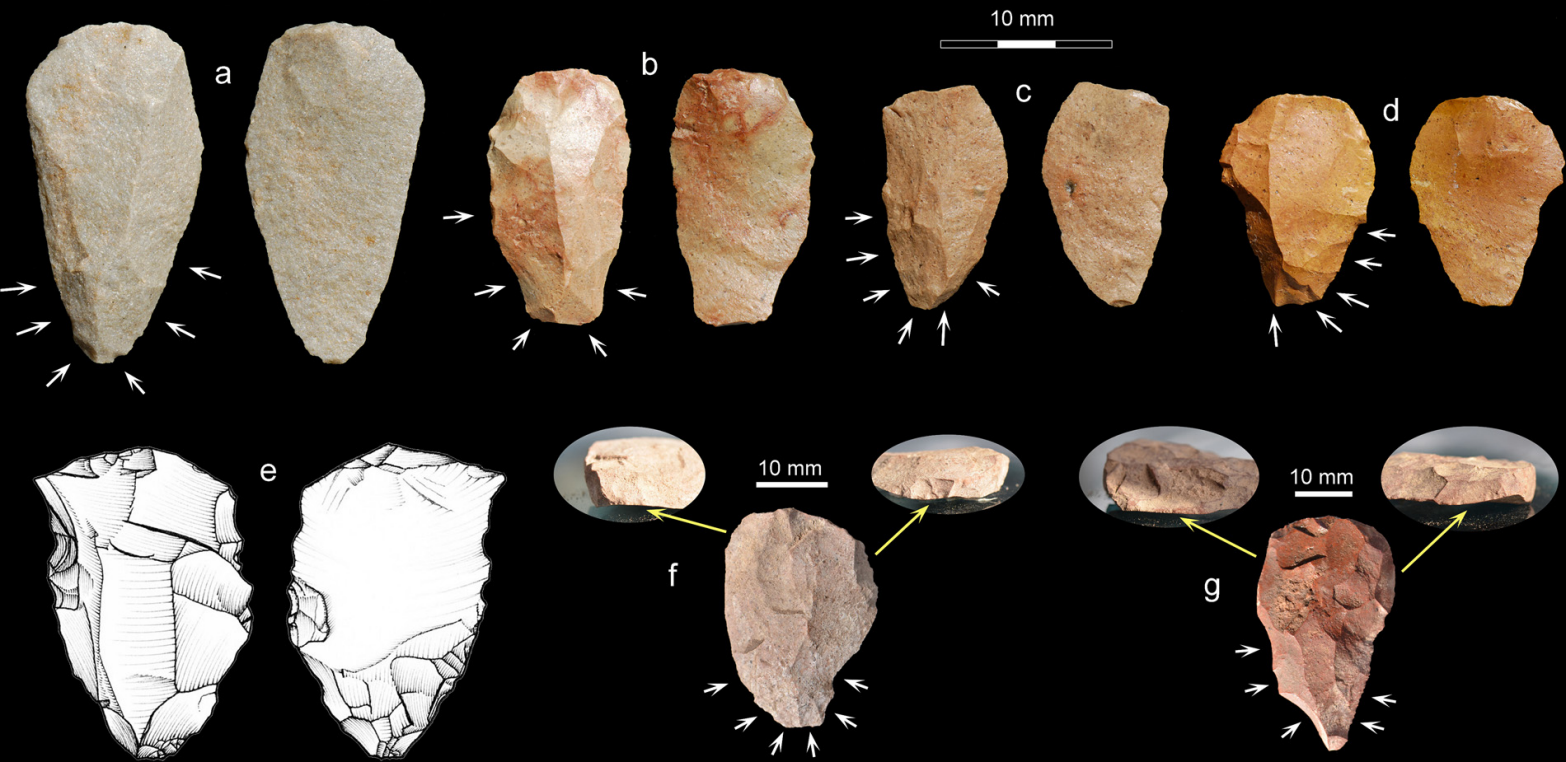
Levallois points with Nubian characteristic from UPK7 and Mertenhof. UPK7: a, quartzite; b-d, silcrete; Mertenhof: f & g, both silcrete. White arrows show directions of preparation removals. Overshot flake from Nubian-like core (e, silcrete). Inset images for Mertenhof points show damage immediately below flake platforms.

In addition to the Nubian cores, products deriving from this reduction system occur at UPK7 (n = 14; Fig 9). These convergent flakes show negatives from type 2 or type 1/2 Nubian preparation with facetted platforms, exterior platform angles close to 90° and feathered terminations on all edges. The mean length of the last removals on the cores´ working surfaces (35.4 mm) attests to the production of predominantly small convergent flakes at UPK7, particularly for silcrete and chert. Furthermore, three overshot flakes preserve the distal and lateral core preparation matching the existence of overshot removal negatives on two of the cores in our sample (Fig 7c). These products confirm the in situ exploitation and subsequent discard of Nubian-like cores.

**Fig 10:**
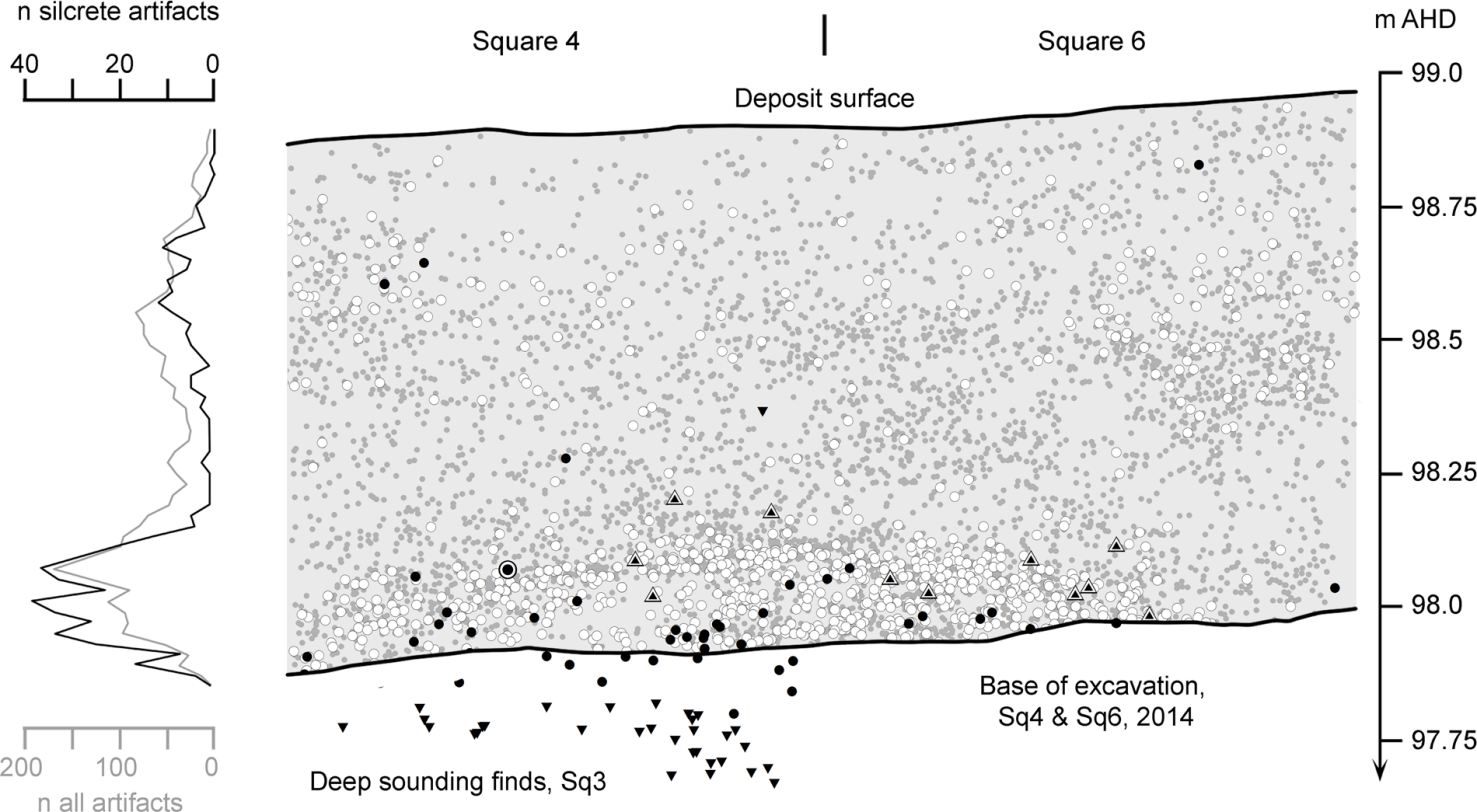
Distribution of artefacts in the Mertenhof sequence. Non-silcrete artefacts (filled grey circles) and silcrete artefacts (white circles) are shown from squares 4 and 6 only (see S2 Text for square locations). Unifacial points (nested triangles), backed artefacts (black circles), bifacial points and thinning flakes (inverted black triangles) are shown from all excavated squares, including the deep sounding square 3. The single nested circle represents a core with Nubian characteristics. Frequency histograms for all artefacts (grey line and scale) and silcrete artefacts (black line and scale) are generated using arbitrary 20 mm horizontal bins.

Situated 25 km south west of UPK 7, Mertenhof Rock Shelter preserves a long sequence of late Pleistocene lithic industries (see S2 Text; S1 Fig). Artefact density shows a distinct peak between 98.14 m and 97.9 m above arbitrary height datum coincident with the shift to stratigraphic unit BGG/WS (Fig 10). The proportion of silcrete also peaks in this range (Table 3). Unifacial points are common through the upper part of BGG/WS and the immediately overlying unit DGS. In contrast, backed microliths are frequent in lower BGG/WS (Table 4; S2 Fig; see S2 Text for further discussion).

**Table 3:** Number of artifacts and percentages of major raw material types by stratigraphic unit from Mertenhof (pit and burial units excluded).

Unit	n	Hornfels	Quartz	Quartzite	Silcrete	Chert	DWS*
ULBD	572	31.6	19.9	36.5	4.4	4.4	0.2
R/GBS	1043	27.6	16.9	35.0	13.1	5.2	0.0
LGS	782	17.1	26.3	35.9	6.1	7.7	4.1
LRS	702	12.5	23.4	37.5	2.4	5.0	14.2
DGS	876	4.5	10.7	44.6	4.7	1.5	29.1
Upper BGG/WS	3227	2.2	4.0	43.9	27.3	3.6	14.6
Lower BGG/WS	2561	3.1	6.1	17.9	32.2	15.2	19.0
RGS	444	3.4	8.3	41.9	15.1	2.3	17.3
DBS	339	4.7	5.9	66.1	1.2	0.0	10.0
*Total (n)*	*10546*	*911*	*1097*	*3795*	*2045*	*701*	*1455*

*DWS = degraded white stone.

**Table 4:** Frequency of major artefact types by stratigraphic unit from Mertenhof (pit and burial units excluded).

Unit	Scrapers	Notched flakes	Backed microliths	Levallois points	Unifacial points	Bifacial points	BTF*
ULBD	7	0	0	0	0	0	0
R/GBS	3	0	1	0	0	0	0
LGS	2	0	0	0	0	0	1
LRS	1	2	1	5	0	0	0
DGS	4	3	0	13	3	0	0
Upper BGG/WS	6	3	5	31	9	0	0
Lower BGG/WS	2	6	36	5	2	0	0
RGS	0	1	2	2	0	5	26
DBS	0	0	0	3	0	0	0
*Total*	*25*	*15*	*46*	*59*	*14*	*5*	*26*

*BTF = bifacial thinning flakes.

The assemblages in the upper parts of BGG/WS—dense, silcrete-rich and containing unifacial points—conform to the characteristics of the very earliest “post-Howiesons Poort” at nearby Klein Kliphuis and Diepkloof, dated 60–50 ka [87,88,90,92]. The so-called “post-Howiesons Poort” is widespread across southern Africa, exhibits a consistent stratigraphic position relative to other industries, and without exception dates to early MIS 3 [93,94] (see also Table 1). The immediately underlying assemblages at Mertenhof are typical of the Howiesons Poort, dating >60 ka [92,95–97]. Bifacial points and associated thinning flakes underlie the distribution of backed microliths and are associated with the Still Bay industry dating >70 ka [92,95,96,98,99].

That the putative post-Howiesons Poort at Mertenhof is situated in an unusually complete late Pleistocene sequence makes it unlikely that it is in fact some other industry. In the sample recovered so far, the post-Howiesons Poort component of Mertenhof has produced one core similar in form to those classified as Nubian, though this lacks installation of a distal platform (Fig 7k). Mertenhof has also produced two unretouched Levallois points that were manufactured from Nubian-like cores, both of which have damage immediately below the platform that may relate to hafting (Fig 9). All three of these artifacts derive from the upper BGG/WS unit.

## Discussion

A total sample of 36 preferential Levallois cores from UPK7, mostly deriving from an area of only 278 m^2^, match the stringent definition of the Nubian as outlined in [58]. The contextual data from Mertenhof and regional sites associate these cores with the early post-Howiesons Poort, confidently age bracketed ~60–50 ka. Several lines of evidence suggest that appearance of Nubian core reduction systems in southern Africa reflects convergence on the systems of north-east Africa and Arabia based on the criteria we outlined at the start of this paper.

The first is the large spatial gap between the northern and southern occurrences of Nubian systems. While such systems are widespread in north Africa and occur as far south as Kenya, we could find no published accounts or artefact drawings of Nubian cores from central Africa [100–104], south-central Africa [105–109], or eastern Africa south of Kenya [75,108,110,111]. Particularly important is the lack of Nubian-like cores from well-excavated and stratified sites with large lithic assemblages such as Kalambo Falls [112], Mumba Cave [111,113], Apollo 11 [114] and Twin Rivers [115]. While this absence of evidence between southern and north-east Africa could in part be due to discovery or sampling bias, a complete lack of reported Nubian cores from a multitude of variable MSA occurrences is striking considering their high recognition value [57] and long research tradition [60].

Second is the temporal gap between the Nubian occurrences. As noted earlier, most available ages place the occurrence of Nubian core reduction systems in north Africa within MIS 5, with examples of isolated cores in non-Nubian assemblages in MIS 4 and early MIS 3. In contrast to the Nubian techno-complex, the post-Howiesons Poort of southern Africa is well-dated and consistently placed in early MIS 3 only. While there is thus potential for very limited overlap with the occurrence of such core types in North Africa, we note that the vast majority of Nubian-like cores at UPK7 are type 2 and type 1/2. Such cores are common in North Africa during the ‘early Nubian techno-complex’, and thus in early MIS 5. The ‘late Nubian techno-complex’ of North Africa is dominated by type 1 cores, which are all but absent from our sample.

Given these points, in order to constitute dispersal or diffusion of the early Nubian from North Africa, our assemblages must reflect transmission of technological information across between 700–3000 generations (allowing for an origin from the late Nubian techno-complex in MIS 5a to the early Nubian techno-complex in MIS 5e), and sustained over 6000 km of diverse environments from the northern deserts through the tropics to the arid and semi-arid regions of the southern temperate zone without leaving an intervening technological signal. If diffusion, this explanation requires high fidelity transmission of that technological system through product copying over 14 000–60 000 years to the exclusion of other technological variants associated with populations encountered en route from south Sudan to the south-western tip of Africa. In order to represent dispersal, this needs to have occurred without leaving a genetic signature, given that “African populations have maintained a large and subdivided population structure throughout much of their evolutionary history” [116], and that genetic studies indicate that north-south dispersals across the various climatic zones and biomes of Africa have been limited during the Pleistocene [116–120]. To that end we note that, while technological industries have occasionally been documented at the continental scale (e.g., Clovis), we are unaware of any industry associated with anatomically modern humans that extends from the temperate zones of the northern hemisphere to those of the southern hemisphere across the tropics. Within Africa, major industries such as the Lupemban, Aterian, Howiesons Poort and Still Bay are restricted to either one temperate zone or to the tropics.

Third, while Nubian-like cores occur in our samples, they are accompanied by unifacial points that are otherwise typical of the post-Howiesons Poort in the region. Thus, our samples and those in the north east of Africa are linked solely by a specific core form.

Overall, given that we have replication of a single technological element between spatially and temporally isolated assemblages, and allowing that the potential sampling interval covers up to 60 000 years across the breadth of Africa, convergence necessarily constitutes the most parsimonious explanation for the Nubian cores found at UPK7 in southern Africa.

Interpreting the UPK7 assemblage as including an instance of technological convergence on the Nubian core reduction system carries several implications. Foremost, the distribution of Nubian cores cannot be assumed to reflect information sharing networks. This does not fundamentally confound the interpretations of [37,57,58] but it does complicate them. In cases where similar lithic systems occur in the same restricted time interval in contiguous areas, information transmission with or without attendant population movement remains a relatively parsimonious explanation. The validity of this hypothesis, however, is contingent on establishing chronological controls for relevant samples, as well as more detailed technological and quantitative comparisons of entire lithic assemblages rather than a single core reduction method. At the moment, such assessments are complicated by a lack of consent regarding different elements of the Nubian or Afro-Arabian Nubian techno-complex and the equation of this unit with a particular group of people [38,59]. In this regard, the recent demonstration of technological convergence of tanged artefacts between North Africa and Arabia—with the latter probably dating to the Holocene—serves as a note of warning [67,68].

More generally, arguments that rely on lithic technologies to track the dispersal of modern human populations across Africa and beyond (e.g., [40]) are necessarily problematic where they assume that the degree of similarity in lithic reduction system informs on the degree of population relatedness in assemblages that are widely separated in space and time [36]. Unrelated populations made similar artefacts [121], and related populations made quite different artefacts given the passage of relatively brief amounts of time [122]. Most problematic in this regard are hypotheses that are based on single core or tool types [37,40,57] which not only mask assemblage variability but also increase the chance of convergence [66,123]. More robust hypotheses may be built using approaches that characterize variability across several lithic domains with a focus on quantitative data and multivariate statistical analyses [35,50,66,123,124]. With presently available data, however, our confidence in lithics as a proxy for the dispersal routes taken by early modern human within and out of Africa must remain weak [34].

## Supporting Information

S1 Fig Mertenhof Rock Shelter. Left panel (a) plan view of topo points on shelter walls and immediate talus (green point), with shelter walls and excavation squares shown in white; b) section view of topo points with plotted finds (blue circles); (c) layout of squares. Right panel shows excavation at the end of season 3.
(TIF)

S2 Fig Sample of artefacts from Mertenhof Rock Shelter. (a-d) platform (bladelet) cores, Robberg layers; (e) truncated quartz blade, (f) chert segment, (g) chert truncated notched blade, Howiesons Poort layers; (h) silcrete unifacial point tip, (i) hornfels unifacial point, post-Howiesons Poort layers; (j) silcrete bifacial point, (k) quartzite bifacial point, Still Bay layers; (l) quartzite denticulate, (m) quartzite blade, early MSA layers. White dots show location of retouch on backed artefacts and denticulates.
(TIF)

S1 Text Nubian core spreadsheet. Example of a filled-in Nubian core spreadsheet with a schematic sketch of the configuration and sequence of removals on the main working surface.
(DOCX)

S2 Text Archaeology of Mertenhof Rock Shelter, Western Cape, South Africa. (DOCX)
