# Supplementary figures and images for: Implications of Nubian-Like Core Reduction Systems in Southern Africa for the Identification of Early Modern Human Dispersals
Source: PLoS One. 2015 Jun 30;10(6):e0131824. doi: 10.1371/journal.pone.0131824 (PMC4488358; doi:10.1371/journal.pone.0131824)

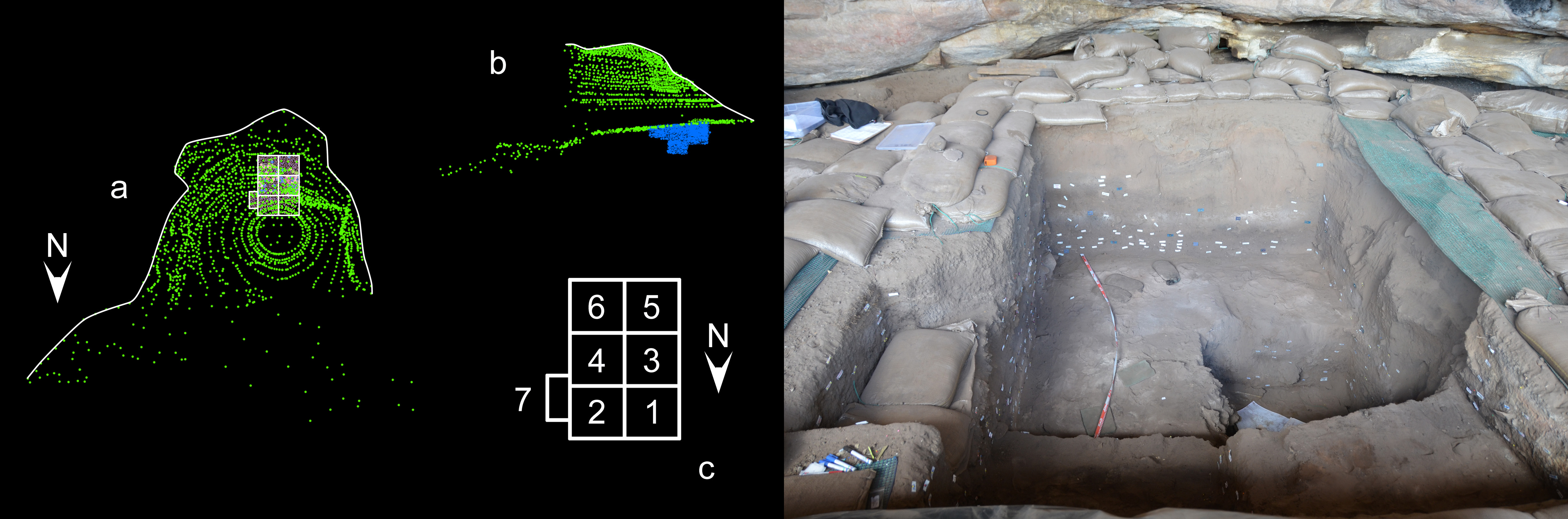

Supplement: S1 Fig — Left panel (a) plan view of topo points on shelter walls and immediate talus (green point), with shelter walls and excavation squares shown in white; b) section view of topo points with plotted finds (blue circles); (c) layout of squares. Right panel shows excavation at the end of season 3. (TIF) [file pone.0131824.s001.tif]

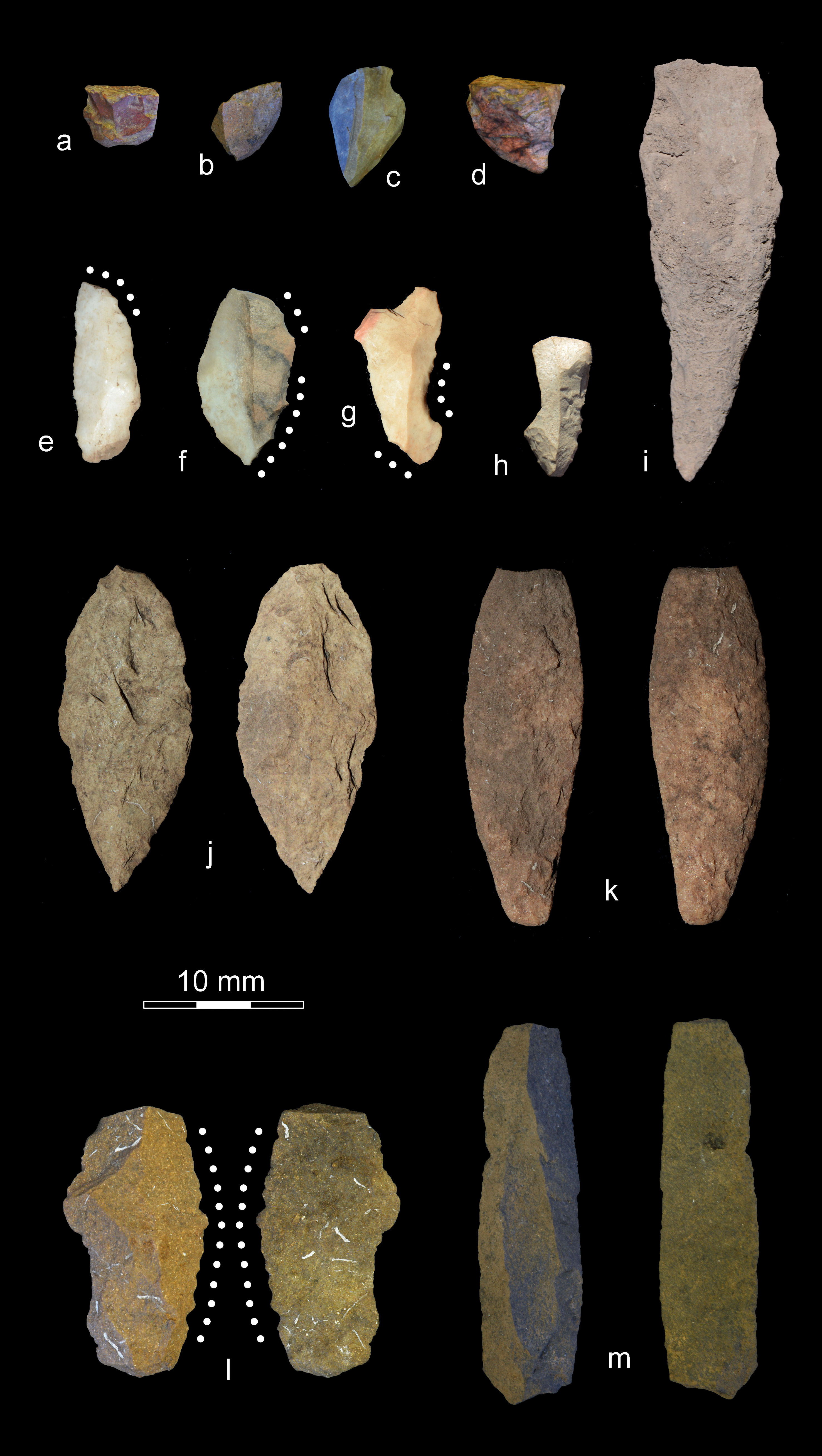

Supplement: S2 Fig — (a-d) platform (bladelet) cores, Robberg layers; (e) truncated quartz blade, (f) chert segment, (g) chert truncated notched blade, Howiesons Poort layers; (h) silcrete unifacial point tip, (i) hornfels unifacial point, post-Howiesons Poort layers; (j) silcrete bifacial point, (k) quartzite bifacial point, Still Bay layers; (l) quartzite denticulate, (m) quartzite blade, early MSA layers. White dots show location of retouch on backed artefacts and denticulates. (TIF) [file pone.0131824.s002.tif]
