# Supplementary material for: Implications of Nubian-Like Core Reduction Systems in Southern Africa for the Identification of Early Modern Human Dispersals
Source: PLoS One. 2015 Jun 30;10(6):e0131824. doi: 10.1371/journal.pone.0131824 (PMC4488358; doi:10.1371/journal.pone.0131824)
Supplement: S1 Text — Example of a filled-in Nubian core spreadsheet with a schematic sketch of the configuration and sequence of removals on the main working surface. (DOCX) [file pone.0131824.s003.docx]

**Supporting information text 1: Nubian core spreadsheet**

**
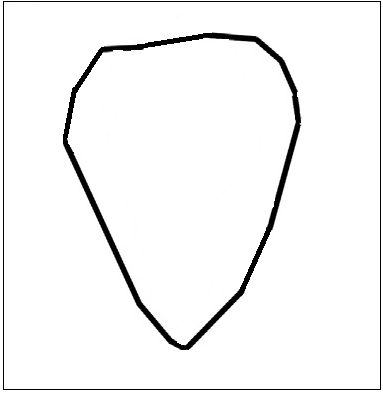
ID:** XXXX  **#Photo:** XXX.jpeg

**Nubian type**

1 2 1/2 bid. 1/2 lat.

early stage indeterminate

**Core morphology**

Triangular Pitched Cordiform

Rectangular Irregular

**Platform preparation**

Facet. Dihed. Plain Cortex

**Distal medial ridge angle**

steep <60° semi-steep 60-90° oblique 90-120° outrepassé Missing

**Distal platform angle Raw material**

Silcrete

right 80-90° semi-acute 80-60° acute <60°

**Morphology of removals Number of removals Aberrants?**

0

1

Point Flake Blade

**Heat-treated? %cortex: cortex type:** fluv. outcr. **Weathering?**

**Weight:** 21.2 g  **MD:** 45 mm **Length:** 43 mm **Width:** 35 mm **Thick.**: 22 mm

**Comments Blank type:** Non-Flake Flake
