# Supplementary material for: Implications of Nubian-Like Core Reduction Systems in Southern Africa for the Identification of Early Modern Human Dispersals
Source: PLoS One. 2015 Jun 30;10(6):e0131824. doi: 10.1371/journal.pone.0131824 (PMC4488358; doi:10.1371/journal.pone.0131824)
Supplement: S2 Text — (DOCX) [file pone.0131824.s004.docx]

**Supporting Information Text 2**

**Convergence in derived elements of lithic technologies complicates identification of early modern human dispersals**

Manuel Will

Department of Early Prehistory and Quaternary Ecology

University of Tubingen

Alex Mackay

Centre for Archaeological Science

School of Earth and Environmental Sciences

University of Wollongong

Natasha Phillips

Centre for Archaeological Science

School of Earth and Environmental Sciences

University of Wollongong

**Archaeology of Mertenhof Rock Shelter, Western Cape, South Africa**

Mertenhof Rock Shelter is situated in a band of Table Mountain Sandstone at the eastern edge of the Cederberg mountains in the Western Cape of South Africa. The shelter faces north and is perched above the Biedouw River on the southern face of the kloof (low canyon) (Figure S1). The sheltered area measures 10 m across the dripline by 9 m from the shelter mouth to the deepest point (Figure SI 2). The site has been excavated under the direction of Aara Welz and Alex Mackay over three seasons in April/May 2013, September/October 2013 and October 2014.

*Excavation methods*

Six 1 m x 1m squares were delimited in a single trench 3 m x 2m (Figure S2). The long axis is oriented grid north, which is towards the shelter mouth. The six squares were numbered 1 to 6, and each was subdivided into four quadrants. A partial seventh square was added to enable recovery of a burial.

Excavation followed natural stratigraphy, with excavation units not exceeding 30 mm depth. In the case of stratigraphic units >30 mm, these were excavated in spits following the last known slope. Archaeological materials were piece plotted using a total station and assigned individual find numbers. Different size cut-offs were employed for different classes of finds: >20 mm for stone artefacts, ochre and ostrich eggshell, >30 mm for bone. In addition all beads (glass, bone and ostrich eggshell) were plotted regardless of size, as were worked pieces of bone and certain classes of stone artefacts such as retouched flakes, cores and bladelets.

The exception to the standard plotting regime occurred in the centrally-located square 3 where, during the first season, only certain finds (beads, worked ochre, retouched flakes, cores and bladelets) were plotted. The objective of this was to expedite excavation in square 3 in the first season to provide a lead (witness) section for the adjacent squares, and to provide a relative rapid assessment of the site sequence and the value of continuing work there past the first season. All squares other than square 3 had all artefacts plotted as per the preceding paragraph, and from the start of season two (September/October 2013) full piece plotting was also applied to square 3. Though square 3 was not excavated in the most recent (third) season it remains the deepest excavated part of the deposit.

*Archaeological and stratigraphic sequence*

All plotted artefacts from the first two seasons of excavation at Mertenhof have been classified, as have all plotted finds from squares 4, 5 and 6 in the most recent season. The relationship between the major stratigraphic units and key artefacts types is shown in Table 4 in the main text body; raw material frequencies are provided in Table 3.

The uppermost unit is ULBD, comprising Upper Loose, Bedding and Dung Crust strata. All relate to late Holocene occupation. Among other finds, the unit includes 58 beads made from ostrich eggshell and bone, and 11 glass trade beads. Quartzite and hornfels are the dominant rocks in this unit, the latter most likely sourced from the Doring River given the prevalence of river cobble cortex and the absence of this material west of the Doring.

The Red and Grey Brown Series (R/GBS) includes numerous small platform and rotated cores made from fine grained rocks such as silcrete and chert, typical of the Robberg industry ([1-3](#_ENREF_1)) (Figure S3). Quartzite and hornfels remain the most common rocks though silcrete is proportionally frequent. R/GBS is truncated by multiple pits, from two of which the poorly preserved remains of at least three small children were recovered.

The Light Grey Series (LGS) and Lower Red Series (LRS) include flakes with faceted and dihedral platforms, indicative of MSA occupation, though only a single discoidal cores has so far been recovered from unit. Other than a few Levallois points and a single backed microlith, no typical markers of known MSA industries occur in these layers and artefact densities are low. The characteristics of MSA assemblages in south western South Africa after 50 ka are poorly known ([4](#_ENREF_4)), and the assemblages from LGS and LRS likely fall within this period. Both LGS and LRS have elevated percentages of quartz but are differentiated in raw material composition by the relative abundance of decayed white stone (DWS), which is more common in the lower unit. DWS frequency is broadly inversely proportional to hornfels frequency, and displays similar texture and cortex to hornfels; it is likely a degraded form of that rock. The difference in DWS frequency between LGS and LRS may reflect some elapse of time between the two units.

The Dark Grey Series (DGS) has very high proportions of quartzite and DWS, and low proportions of all other rocks. In addition to Levallois (n=3) and discoidal (n=1) cores, DGS has reasonably large numbers of unretouched Levallois points and three unifacially retouched points. The unit matches the characteristics of the later post-Howiesons Poort from nearby Klein Kliphuis ([5](#_ENREF_5)).

The Brown Grey Grit and White Silt (BGG/WS) unit is subdivided into upper and lower units based on relative stratigraphic position and artefact composition. The upper unit has elevated proportions of quartzite and silcrete and low values for all other raw materials. Upper BGG/WS includes 31 Levallois points, nine unifacial points and five backed microliths. The unit also includes the Nubian-like core and both Levallois points with Nubian characteristics discussed in the Main Text. All three of these artifacts derive from the lowest strata in upper BGG/WS in contexts where both backed microliths and unifacial points occur together. The co-occurrence of these artefact classes is also noted in the main analytic area at UPK7 (Main Text, Methods). The characteristics of lower BGG/WS – with elevated proportions of silcrete and quartzite, and the presence of both backed microliths and unifacial points – match the characteristics of the earliest post-Howiesons Poort layers at Klein Kliphuis and Diepkloof ([5](#_ENREF_5), [6](#_ENREF_6)).

Lower BGG/WS has the highest proportions of silcrete and chert in the Mertenhof sequence, and the lowest frequency of quartzite. To that extent, lower BGG/WS is quite distinct from the upper component of the same unit. Lower BGG/WS contains both backed microliths and unifacial points, though the relative frequency of these types is reversed; backed microliths are abundant and unifacial points are uncommon. Unretouched Levallois points are also much less frequent in lower BGG/WS. More common are flakes and blades with complex notches similar to those observed in Howiesons Poort contexts at sites such as Diepkloof, Klipdrift and Pinnacle Point 5/6 ([6-8](#_ENREF_6)). Overall, the assemblage from lower BGG/WS is typical of known Howiesons Poort occurrences across southern Africa ([1](#_ENREF_1)).

In the underlying Red Grey Series (RGS), silcrete proportions drop considerably and backed microliths become infrequent. Bifacial points and bifacial thinning flakes are, however, reasonably common (Figure S3). While arguments have been presented for an extended duration of bifacial point production at some sites in north eastern South Africa, at sites within 1000 km of Mertenhof, bifacial points tend to occur in a single coherent band, which is conventionally referred to as the Still Bay ([6](#_ENREF_6), [9](#_ENREF_9), [10](#_ENREF_10)).

The deepest parts of the excavation (Dark Brown Series - DBS) generally lack retouched flakes and are dominated by quartzite to a greater extent than any other unit. These characteristics are typical of the early MSA in the region ([11](#_ENREF_11)).

In summary, the MRS sequence include late Holocene, Robberg, late MSA (possibly two variants), early and late post-Howiesons Poort, Howiesons Poort, Still Bay and early MSA units. As such this appears to be one of the most complete late Pleistocene cultural sequences presently known in southern Africa.

**References**

1. Mackay A, Stewart BA, & Chase BM (2014) Coalescence and fragmentation in the late Pleistocene archaeology of southernmost Africa. *Journal of human evolution* 72:26-51.

2. Lombard M*, et al.* (2012) South African and Lesotho Stone Age sequence updated. *South African Archaeological Bulletin* 67(195):120-144.

3. Deacon J (1982) The Later Stone Age in the southern Cape, South Africa. PhD Phd Thesis (University of Stellenbosch).

4. Mackay A*, et al.* (2014) Putslaagte 1 (PL1), the Doring River, and the later Middle Stone Age in southern Africa’s Winter Rainfall Zone. *Quaternary International* 350:43-58.

5. Mackay A (2010) The Late Pleistocene archaeology of Klein Kliphuis rockshelter, Western Cape, South Africa: 2006 excavations. *South African Archaeological Bulletin* 65(192):132-147.

6. Porraz G*, et al.* (2013) Technological successions in the Middle Stone Age sequence of Diepkloof Rock Shelter, Western Cape, South Africa. *Journal of Archaeological Science* 40(9):3376-3400.

7. Brown KS*, et al.* (2012) An early and enduring advanced technology originating 71,000 years ago in South Africa. *Nature* 491(7425):590-593.

8. Henshilwood CS*, et al.* (2014) Klipdrift Shelter, southern Cape, South Africa: Preliminary Report on the Howiesons Poort layers. *Journal of Archaeological Science* 45:284-303.

9. Villa P, Soressi M, Henshilwood CS, & Mourre V (2009) The Still Bay points of Blombos Cave (South Africa). *Journal of Archaeological Science* 36(2):441-460.

10. Evans U (1994) Hollow Rock Shelter, a Middle Stone Age site in the Cederberg. *Southern African Field Archaeology* 3:63-73.

11. Volman TP (1980) The Middle Stone Age in the Southern Cape. PhD (University of Chicago).
